# Early Onset of Lung Cancer in Small Areas as a Signature of Point Pollution Sources

**DOI:** 10.3390/cancers16061189

**Published:** 2024-03-18

**Authors:** Ettore Bidoli

**Affiliations:** Cancer Epidemiology Unit, IRCCS Centro di Riferimento Oncologico, 33081 Aviano, Italy; bidolie@cro.it; Tel.: +39-0434-659354; Fax: +39-0434-659231

**Keywords:** lung cancer, early onset, point sources of pollution, small-scale area

## Abstract

**Simple Summary:**

At a small-area scale, the detrimental effect of air pollution on lung cancer is challenging to identify and measure due to the potentially scattered detection of pollutants and/or limited statistical power of available indicators. A novel method is presented to detect and quantify the detrimental impact of pollution in small areas by employing the early onset of lung cancer as a signature of pollution. Early-onset lung cancer may speed up the investigation of potential environmental hazards in a specific area, enabling public health officials or citizen committees to carry out geographical analyses.

**Abstract:**

The impact of air pollution on lung cancer (LC) is difficult to detect in low-populated areas due to the potentially unfocused detection of pollutants and/or limited statistical power. This study identified and measured the harmful effect of pollution in small areas by considering the early onset of LC as a signature of pollution. This novel method requires a Bayesian standard curve calculated from the median age at LC onset and the corresponding median age of reference populations. Similar medians gathered from the area/s under investigation permits a probabilistic comparison with the standard curve. Statistically significant divergences can be interpreted as early or late LC onset. The method is exemplified in the Trieste municipality (northeast Italy) using data from the Friuli Venezia Giulia Cancer Registry (study population) and from the International Agency for Research on Cancer (reference population). Early LC onset has been observed near the pollution sources. Within 600 m of the iron foundry, onset ranged between 3.2 and 7.7 years earlier in men and between 11.7 and 16.8 years earlier in women. Near the shipyard, early onset was around 4 years in men and 7 years in women, while in the industrial area, early onset was 5 years in women only. Examining early LC onset may speed up the investigation of potential environmental hazards.

## 1. Introduction

Outdoor air pollution and particulate matter from outdoor air pollution are classified as lung carcinogens in humans (Group 1) by the International Agency for Research on Cancer (IARC) [1,2]. Nonetheless, at a small-area scale, the detrimental effect of air pollution on lung cancer (LC) is tricky to detect and quantify due to the potential unfocused detection of pollutants and/or the limited statistical power of indicators [3,4].

One difficulty is delineating the geographical distribution of both measured and unmeasured gaseous and particulate pollutants harmful to health [5,6,7,8]. Typically, the distribution of a limited number of specific pollutants is inferred from satellite imagery grids [9]. Alternatively, it is estimated by interpolating data from a small number of fixed monitoring stations, which are not always located near the pollution sources [10,11]. The few estimated pollutants are then considered to be representative of the overall historical and current pollution burden [6]. Otherwise, when pollution is endemic, looking backwards at the natural history of LC timeline may give a metric of pollution exposure. It is well-known that the onset of LC cases at an unusually early age can suggest a past common exposure when the age structure of the population in which LC cases occur is available [12,13]. Consequently, after allowance for all LC risk factors, it is reasonable to exploit an early age at LC onset as a telltale sign of exposure to harmful pollutants.

The second issue is measuring the impact of pollution on LC in small areas. The current methods focus on two approaches: (1) proportional hazards (i.e., relative risks or rate ratios) that measure the strength of an association but may be tarnished by the low statistical power of sparse cases or unmeasured confounding factors [14]; (2) indicators of accelerated failure time (AFT) to cancer incidence or death (i.e., measures of life expectancy, gain or loss of years lived in a definite population, or survival), which require an arbitrarily chosen upper reference age [15,16]. Otherwise, calculating the probability of observing early-onset LC in relation to a reference population outweighs the limitations of the aforementioned indicators.

This study aims to detect probabilistically small areas with LC cases linked to pollution and to estimate the harmfulness of the pollution using a novel derivation of a simple two-term Bayesian linear regression model that takes into account confounding factors. Two metrics were calculated for each area: (1) the number of early or late years to expected LC onset; (2) its posterior probability (PP). This approach is exemplified in a case study conducted in the Trieste municipality (northeast Italy), where three persistent anthropogenic point sources of air pollution have been spotted [17,18,19,20,21]. All incident LC cases for the period of 1995–2013 were reanalyzed by dividing the Trieste municipality into 41 sections instead of 3 main sections [21]. A reference population was extracted from the worldwide LC incidence data built by the International Agency for Research on Cancer (IARC) for the period of 2008–2012 [22].

## 2. Methods

This population-based observational study included all incident LC cases (International Classification of Diseases, 10th edition:C33-34) from two validated data sources.

### 2.1. Datasets

#### 2.1.1. LC Cases and Reference Population

The gathering and inclusion criteria of this dataset have been described previously [23,24]. Briefly, LC data were extracted from the publicly available Cancer Incidence in Five Continents, Volume XI [22]. The volume collected cancer incidence data from 2008 to 2012. The data were available from 393 population-based, country-specific, and region-specific high-quality Cancer Registries (CRs) located in 65 countries worldwide (approximately one-sixth of the world population). The corresponding resident populations from which the cases were drawn were gathered from the same IARC database. The cases and population data were divided by sex, age (0–4, 5–9, …, 85+ years), and CR. All Italian CRs (N = 36) were extracted from this dataset and labeled as the reference population.

#### 2.1.2. LC Cases and Population of the City of Trieste

**Figure 1:**
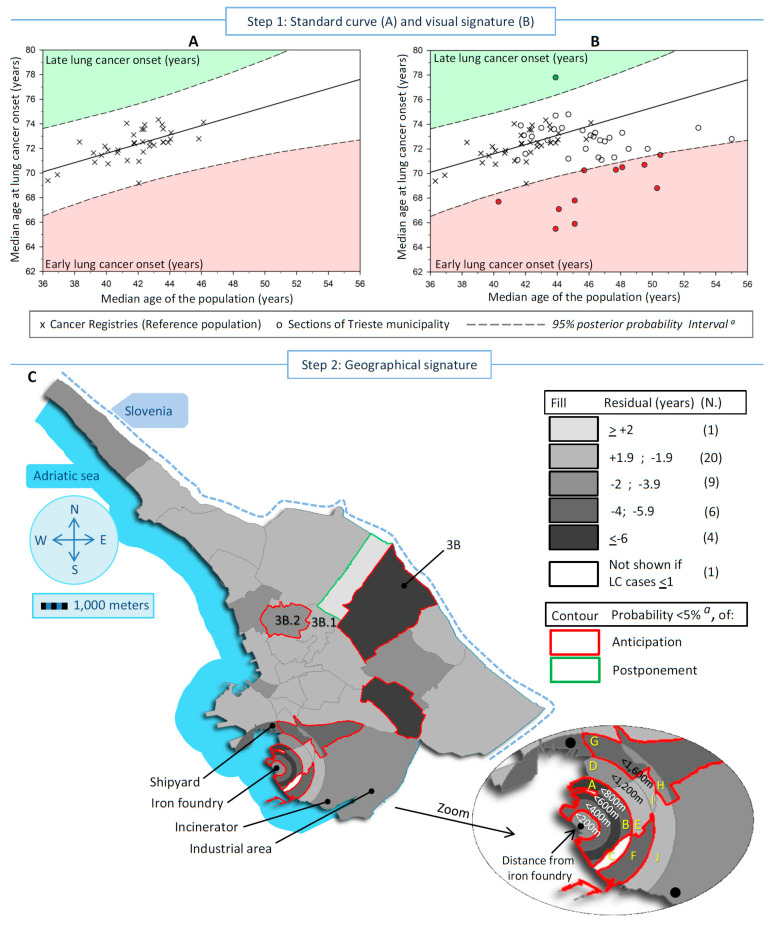
Detection and measure of the excesses of lung cancer (LC) incidence linked to pollution in low populated areas, exemplified in a case-study conducted in Trieste municipality (north-eastern Italy) during 1995–2013, in men (**A**) Standard curve with the scatter plot, linear regression and corresponding 95% posterior probability Intervals ^a^, of the median age at LC onset in the reference populations ^b^ vs. the corresponding median age. Red sector identifies the areas with an early age at LC onset and the green one a late onset. (**B**) Previous scatter plot overlapped with the medians scatter plot (LC onset and population) of the studied sections. Dots outside the 95% intervals are the divergent areas. (**C**) Map of the residuals (observed minus expected age at LC onset), and corresponding posterior probability ^a^ (<5%) of divergence from expectation according to all 41 sections of Trieste municipality. ^a^ Corrected for Bonferroni adjustment for multiple comparisons (probability threshold = 0.00125); ^b^ All Italian Cancer Registries included in the XI volume on Cancer in Five Continents; The letters in yellow represented the specific sections cited in Table 1.

**Figure 2:**
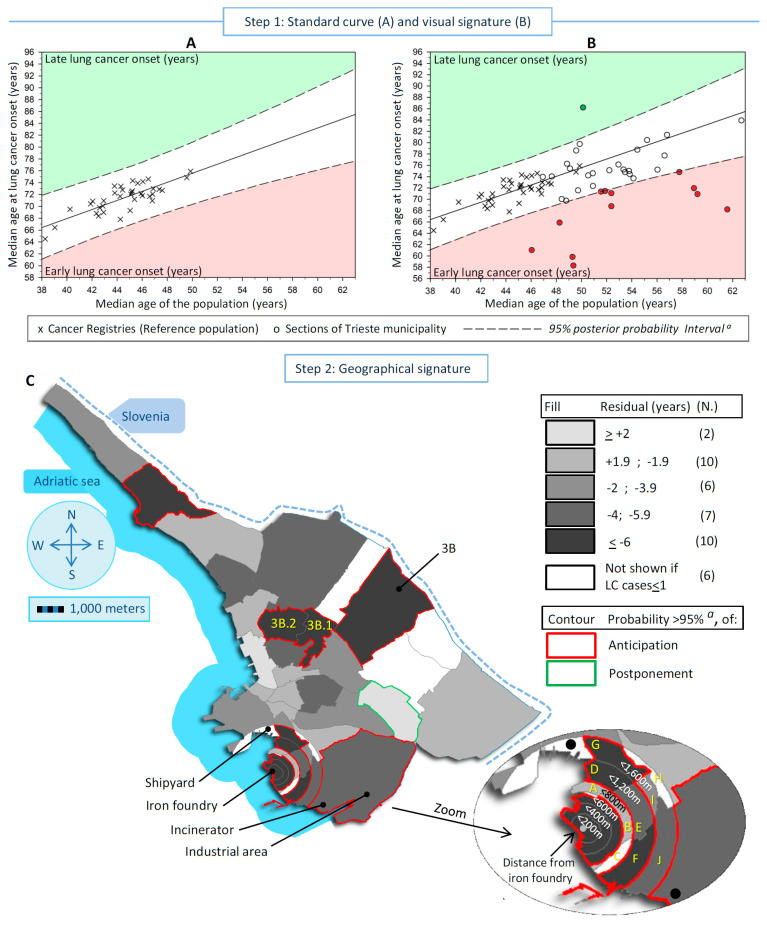
Detection and measure of the excesses of lung cancer (LC) incidence linked to pollution in low populated areas, exemplified in a case-study conducted in Trieste municipality (north-eastern Italy) during 1995–2013, in women (**A**) Standard curve with the scatter plot, linear regression and corresponding 95% posterior probability Intervals ^a^, of the median age at LC onset in the reference populations ^b^ vs. the corresponding median age. Red sector identifies the areas with an early age at LC onset and the green one a late onset. (**B**) Previous scatter plot overlapped with the medians scatter plot (LC onset and population) of the studied sections. Dots outside the 95% intervals are the divergent areas. (**C**) Map of the residuals (observed minus expected age at LC onset), and corresponding posterior probability ^a^ (<5%) of divergence from expectation according to all 41 sections of Trieste municipality. ^a^ Corrected for Bonferroni adjustment for multiple comparisons (probability threshold = 0.00143); ^b^ All Italian Cancer Registries included in the XI volume on Cancer in Five Continents; The letters in yellow represented the specific sections cited in Table 1.

The city of Trieste, with a population of around 200,000 inhabitants, hosts three major pollution point sources. These sources include a shipyard, an iron foundry (decommissioned in 2020), and an incinerator that was replaced by a waste-to-energy plant in 2000. These pollution sources have been previously described in five studies [17,18,19,20,21]. To study the point pollution sources, Trieste was partitioned into 41 areas. The areas were defined geographically a priori according to the following criteria: (1) 18 areas were derived from 5 city neighborhoods fragmented into portions of concentric circles around the iron foundry with increasing radii of 200 m, 400 m, 800 m, 1200 m, and 1600 m; (2) the remaining 23 areas corresponded to the rest of the city neighborhoods. Step 2 in Figure 1 and Figure 2 display the 41 sections, the locations of the three known sources of pollution, the industrial area, and the supplementary area identified in this study.

All 3670 LC incident cases diagnosed during 1995–2013 (2505 men and 1165 women) and collected by the population-based Friuli Venezia Giulia CR (CR-FVG) were considered, i.e., from the beginning of the cancer registration to the end of the availability of georeferenced cases (year 2013). The corresponding 2004 resident population (mid-population of the period 1995–2013) was collected by the regional healthcare population database. For each examined section, the total number of incident cases, the median age at LC onset, the total number of the population, and the median age of the population were obtained from the CR-FVG and the population database.

### 2.2. Statistical Analyses

Two previous papers [23,24] have examined 20 different incident cancers gathered by all 393 worldwide CRs [22]. CRs were idealized by means of two geometrical points, the median age of the population, and the median age at cancer onset. In the IARC dataset, the ages were grouped by quinquennia (0–4, 5–9, …, ≥85 years). Thus, the medians were estimated by means of linear interpolation [25]. Briefly, when LC cases and the corresponding populations were scatter-plotted, the two medians followed a linear pattern. This pattern paved the way for the detection of LC cases linked to pollution in small communities by following two key steps.

**Step 1: Standard curve and visual signature**

The standard curve was based on a reference population that consisted of all Italian CRs (N = 36) [22]. These 36 CRs covered administrative populations approximately ranging from 60,000 to 1,300,000 inhabitants in each sex. The choice of this reference population was essentially dictated by: (1) sharing the Italian smoke-free law (10 January 2005); (2) none of the 36 CRs as a whole were affected by an anthropogenic pollution pattern similar to each section of the Trieste municipality; and (3) the affiliation to the Italian Association of Cancer Registries (AIRTUM), which has promoted shared approaches to cancer registration and the same rules of cancer coding across Italy since 1996 (https://www.registri-tumori.it/cms, accessed on 15 January 2024).

The standard curves were calculated by means of a simple Bayesian linear regression model, including a predictor variable (i.e., the median age of the population in each CR) and a dependent variable (i.e., the median age at LC onset in the corresponding population) and displayed in Figure 1A and Figure 2A (men and women, respectively). Each CR is visualized as an “X” sign. The regression line allowed for the calculation of the expected age at LC onset in additional populations when their median age was known. The difference (residual) between the observed age at LC and the expected value gives the years of early-onset (negative difference) or late-onset (positive difference) LC. The residual can be associated with a risk factor [12,13], i.e., the pollution signature, after allowance for the other risk factors of LC. The key issue is to discriminate the genuine early/late onset of LC from that arising purely by chance or confounding.

The effect of pollution can be overwhelmed by the interference of a number of risk factors associated with LC (confounding factors), including active smoking (the leading risk factor for LC), prevalence of EGFR mutation [26], second-hand smoking, engine exhaust, radon exposure, obesity, occupation, type of household heating and cooking, and deprivation status [27]. In addition, the exposure level, the prevalence, and the start and duration of exposure to each risk factor has fluctuated substantially worldwide [28]. Consequently, the varying effects of these risk factors fostered the number of LC cases, their age distribution, and thus, the level of dispersion of CRs within the scatter plots displayed in Figure 1A and Figure 2A. The observed dispersion led to an indirect separation of pollution (characterized by the anticipated age of LC onset) from the confounders. In particular, the dispersion can be restrained within a priori specified lower and higher bounds of a 95% posterior probability interval (PPI) derived from the Bayesian linear regression. PPI bounds are the interval in which to expect new observations to be found with a 95% probability, given what is observed in the reference population. The outlier sections outside the 95% PPI bounds indicate the pollution signature: the downside zone corresponded to early LC onset (filled in red), and the upside zone (filled in green) to late onset (Figure 1A and Figure 2A).

Lastly, the 41 study areas are idealized by their median ages (at LC onset and of the population) in a second scatter plot, which was overlapped and compared with the standard curve (Figure 1B and Figure 2B) to check if any section lies outside the 95% PPI bounds. Each section is represented by a circle.

**Step 2: Geographical signature**

In addition to computing residuals, we used a Bayesian quantitative approach to compute the posterior probability (PP) that any observation was an outlier compared to the standard curve, i.e., if a residual was significantly different from 0. This hypothesis testing approach considered the multiple testing issue, and a Bonferroni correction was applied a priori to the alpha threshold. Alpha (equal to 0.05) was divided by the number of areas with more than 1 incident case (40 in men and 35 in women). Therefore, the threshold used was 0.00125 (0.05/40) in men and 0.00143 (0.05/35) in women.

The residual and the PP of early/late-onset LC were mapped into the 41 sections of the Trieste municipality, separately for men and women. The areas are colored in shades of gray that represent the residuals grouped into five categories (<+2 years postponement—lighter gray, +1.99 to −1.99 years, −2 to −3.99 years, −4 to −5.99 years, and ≥6 years anticipation—darker gray) and bordered in red when anticipation was statistically significant or in green when the postponement was statistically significant. The white-colored areas are those with an observed number of cases ≤1 during the whole period examined.

A major concern of this study was the potential influence of the reference population in the computation of the early/late onset of LC and its PP of occurrence. To support the strength of this study, a sensitivity analysis was carried out using a different reference population, i.e., all Italian CRs located in the Po Valley (N = 23). These CRs share a smog blanket with the Trieste municipality [29].

### 2.3. Statistical Software

All statistical analyses were carried out by WinBUGS (v. 1.41), a public domain package for Bayesian inference using Markov Chain Monte Carlo (MCMC) methods. The first 100,000 samples of 3 chains were discarded as burn-in, and the following 100,000 iterations were sampled (running length) and used in the present study. The software SAS (v. 9.4 SAS Institute, Cary, NC, USA) computed the frequencies. Scatter plots with linear regression lines and 95% PPIs were drawn using SigmaPlot (v.11), while maps were drawn by SAS.

## 3. Results

During the 1995–2013 period, the incident LC cases observed in the Trieste municipality were 2435 men and 1122 women. The number of cases varied in each section, ranging from 0 to 287 in men and from 0 to 109 in women. The population in these sections ranged from 4 inhabitants to 10,929 in men and from 3 to 11,940 in women. In the 36 Italian CRs included in the reference population, the number of cases varied from 57 to 1476 per year in men and from 20 to 656 in women. The population in these CRs ranged from 77,938 inhabitants to 1,319,795 in men and from 64,802 to 1,432,957 in women.

Figure 1 and Figure 2 show, in both sexes, the steps to detect and localize the sections of the Trieste municipality linked to pollution. Figure 1A and Figure 2A display the standard curves, with the scatter plots representing the median age at LC onset in the Italian CRs in the vertical axis vs. the median age of the population in the horizontal axis, plus the linear regression that fits the two medians and the 95% PI. The equations for the linear regression were:

in men: [median age at cancer onset] = 56.55 + 0.38 × [median age of the population]; *r*^2^ = 0.41

in women: [median age at cancer onset] = 37.40 + 0.76 × [median age of the population]; *r*^2^ = 0.64

For men, the median age at LC onset increased by about one-third of a year for each one-year increase in population aging, while in Italian women, the increase was about three-quarters of a year.

In Figure 1B and Figure 2B, the standard curves overlap with the scatter plot of the studied sections idealized by their medians (LC onset and population). In men, 12 sections were outside the 95% PI, of which 11 displayed early onset of LC, and 1 late onset. In women, 12 sections showed early LC onset and 1 late onset. Finally, Figure 1C and Figure 2C display maps of the residuals of the 41 studied sections to identify the areas at risk. In men, the statistically significant divergent sections with early LC onset were near the iron foundry (more than 2 years earlier up to 800 m from the source of pollution), near the shipyard (between 2 and 3.9 years), and in a bordering eastern section in the center of the map (more than 6 years earlier). In women, the statistically significant divergent sections were near the iron foundry up to 1200 m (over 4 to 6 years earlier of LC onset), plus an area located between 1200 m and 1600 m near the shipyard, the shipyard (more than 6 years earlier), in the industrial area (between 4 and 5.9 years earlier) where the waste-to-energy plant was located, and in the same bordering eastern described before for men (more than 6 years earlier). A total of 7 sections out of 41 shared an earlier onset of LC between sexes.

Table 1 shows the number of LC cases and residents, the residuals with the corresponding 95% credibility interval (CI), and the PP of the residuals. The PPs were considered statistically significant after Bonferroni correction (probability thresholds of ≤0.00125 in men and ≤0.00143 in women). Specifically, the portion of circles around the iron foundry showed a statistically significant earlier onset of LC in both sexes up to 600 m, except for men in the circle of a 200 m radius. In particular, below 200 m, the onset of LC was 3.2 years earlier in men (PP = 3.10^−3^, not significant) and 11.7 in women (PP = 10^−5^). In the 200–399 m range, the onset was 4.5 years earlier in men (PP = 2.10^−4^) and 10.4 in women (PP = 10^−5^), and in the range of 400–599 m, the onset of LC was 7.7 years earlier in men (PP = 10^−5^) and 16.8 in women (PP = 10^−5^). Between 600 m and 1,199 m, early onset was distributed heterogeneously across the sections, and only one section displayed a consistent statistically significantly earlier onset of LC in both men (4.0 years; PP = 10^−5^) and women (8.4 years; PP = 10^−6^). Concerning the shipyard, which is located in the range of 1200–1599 m from the iron foundry, a borderline significantly earlier LC onset of 4.4 years (PP = 0.0017) was observed in men, whereas 1 LC case out of 75 inhabitants/year was observed in women, and the median age at LC onset was not computed. Conversely, a statistically significant earlier onset of LC was observed in a neighboring section of the shipyard (section “G” in the range of 1200–1599 m from the iron foundry) both in men (4.1 years; PP = 2.10^−4^) and women (6.7 years; PP = 8.10^−4^). In the industrial area, a 5.3-year earlier anticipated LC onset was seen in women only (PP = 0.0013). Finally, a bordering eastern section (Section 3B) showed a statistically significant earlier LC onset in men of 7.6 years (PP = 10^−5^, eight incident LC cases) and 11.6 years (PP = 10^−5^, three incident LC cases) in women. Two neighboring sections of Section 3B (3B.1 and 2B.2) showed statistically significantly earlier onset of LC in women, while in men, the divergence concerned only Section 3B.2. In the seven sections that shared between the sexes an earlier onset of LC, the difference between the observed median age at LC onset in men and women was 0.4 years, while in the other sections, the median difference was 2.2 years.

## 4. Discussion

This study shows, for the first time, that a novel derivation of a simple Bayesian linear model can successfully determine point sources of pollution at a small-scale level by quantifying the early onset of LC. This approach can be qualitative, by visually comparing the standard curve with the median age of LC cases and of the population in the small areas to investigate; and/or quantitative, by calculating the exact probabilities of occurrence of early-onset LC. The impact of pollution was estimated from a single cross-sectional measure without requiring additional information about any pollutant concentration or about LC confounders due to the availability of a reference population. The early onset of LC can also be interpreted as the years of life lost free from LC. This indicator may be useful in communicating a metric about the health impact posed by air pollution.

Specifically, the estimated number of years of healthy life lost within a 600 m radius of the iron foundry ranged between 3.2 and 7.7 years in men and between 11.7 and 16.8 years in women. Although the impact was somewhat less pronounced around the shipyard, the onset of LC was approximately 4 years earlier in men and 7 years earlier in women. In the industrial area that hosted the incinerator/waste-to-energy plant, only women showed a 5-year early onset of LC. Finally, a harmful effect was observed in a section located at least 5 km from the three main investigated areas, which had never been described before. The age at LC onset in this area was 4.6 years earlier in men and 11.6 years earlier in women. If independently confirmed [30], the simple novel approach used could serve as an additional tool for environmental epidemiology.

These findings align closely with the results of four investigations conducted in the municipality of Trieste, which is characterized by persistent anthropogenic sources of air pollution, including an iron foundry that was decommissioned in 2020, a shipyard, and a waste-to-energy plant that replaced an incinerator in 2000 [17,18,19,20,21]. However, these studies had limited spatial resolution since it was only by combining the populations of several city neighborhoods that a moderate increase in the risk of pollution-related LC emerged. In contrast, the present study employed a higher spatial resolution, allowing for a more focused examination of the sections surrounding the specific point sources of pollution. Moreover, this innovative approach at a finer scale revealed a previously unidentified area at risk, which warrants further investigation.

The findings of this study are consistent with two previous research papers that examined the accelerated time to death from LC in relation to air pollution. However, the magnitude of the effects was lower in those studies. In one study [31], the worldwide reduction in life expectancy from any cause was estimated to be 2.9 years (range of 2.3–3.5). In Europe as a whole, the average reduction in life expectancy was 2.2 years, but the range of variation in life expectancy was not provided. Another study focused on the accelerated risk of LC death based on smoking status [32]. The author outlined a method for calculating the anticipated years of death according to a relative risk. For instance, assuming a relative risk of 1.5 due to pollution, a non-smoker with a LC death occurring at the age of 60 years would have an anticipated shorter life by 4.4 years, while for smokers, it would be 3 years. The divergence in the magnitude of the effect of pollution between these studies and the present investigation could be attributed to the fact that the current study examined areas located in close proximity to pollution sources and potentially included some LC cases employed in the investigated plants.

Finally, Section 3B hosted, from 1950 to 1970, a municipal landfill within the calcareous dolines. However, the association between this landfill and the early onset of LC goes beyond the purpose of this observational study.

Nonetheless, the results of this study indicate that the health benefits of reducing pollution are likely much greater than previously assumed. Finally, the Bayesian approach was used to compute the probability of observing a divergent onset from a referent population. Other methods are available to evaluate this divergence, for instance, Cook’s distance, dffits, or studentized residuals. However, the purpose of this study was to calculate a probability of divergence rather than obtaining the number of standard deviations from an expected value.

The described approach is twofold. Firstly, once the median of any population and the corresponding median age at LC onset are known, the standard curve rapidly gives a threshold probability of observing an earlier onset of LC that is useful for citizen committees that check hazards in specific areas. Secondly, a more comprehensive approach is to map the early onset of LC and its corresponding probability of occurrence to explore the spatial pattern of risk and to identify areas which need further enquiries.

Several strengths of this investigation should be considered. This study was population-based and included all cases and the resident population of the whole municipality of Trieste. The studied sections of Trieste were defined a priori. The used approach allowed for comparing the standard curve with the studied sections both visually, as a qualitative check, and by calculating the probability of early/late LC onset. The linear model solely included two variables gathered from validated and routinely collected data (incidence and population numbers), and the allowance for confounding factors was derived from the reference population. This simple model addressed sparse data in order to detect the effect of pollution in the immediate proximity of the point sources, and to capture rapidly emerging hazards. The ease of calculating the medians and the absence of a custom, crucial code allowed for testing the findings’ applicability to other small settings. Nearly 7% of the LC cases in the 1995–2009 period moved their residence across the Trieste municipality in the 15 years before diagnosis [21]; therefore, this study gives clues about long-term exposure to pollution. Finally, the sensibility analysis that incorporated only the 23 CRs located in the highly polluted area of the Po river valley confirms the observed associations.

Conversely, this study suffers from some limitations common to other observational studies. Firstly, the scatter plots’ dispersion of points offers an indirect means to differentiate the impact of pollution from other LC risk factors. However, this method inherently lacks the resolution to definitively isolate the effects of specific harmful pollutants or to determine their concentration thresholds within a potentially complex mixture of particles, vapors, and gases that can also vary over time [1]. Despite potential spatial variations within sections, this study initially assumed uniform pollutant exposure for all residents as a basis for an initial analysis. As the study relied on LC onset timing for harmful exposure estimation, identifying specific harmful concentration thresholds for the pollutants fell outside of the scope of this work. Secondly, the study addressed confounding factors indirectly through the standard curve method described in the Materials and Methods section. As various LC risk factors have been described and vary worldwide [26,27,28], the standard curve and its corresponding posterior prediction interval (PPI) capture this variability across the different areas. In this study, the reference population included cancer registries not affected by an anthropogenic pollution similar to the studied sections of the Trieste municipality. By overlaying the studied sections onto the standard curve, points falling outside the 95% PPI (outliers) were interpreted as representing earlier- or later-than-expected ages of cancer onset. Early-onset LC cases can suggest shared past exposure [12,13]. In this case, these outliers likely reflect the influence of a risk factor not present in the reference population—the suspected harmful pollutants. Thirdly, this study focused solely on residential exposure to pollution, neglecting potential contributions from commutes to work or leisure activities. Including commuting patterns in future studies with larger participant pools and longer observation periods could provide a more nuanced understanding of population-wide exposure dynamics. Nevertheless, ignoring commuting should flatten the risk estimates if destination is random with respect to pollution. Fourthly, due to privacy concerns, georeferenced data were only available up to 2013. Consequently, this analysis excludes nine years of incidence data (2014–2022). Fifthly, while the low number of cases near the foundry prevented a detailed analysis by histopathological features, a previous study in Trieste [21] found no consistent link between the foundry and specific types of lung cancer, including adenocarcinomas and squamous cell carcinomas. Lastly, **due to the population-level design of the study, no individual risk assessments were feasible.**

## 5. Conclusions

Though this study focused solely on the timing of LC onset and did not delve into specific pollutant levels or air quality standards, calculating the occurrence probability of early-onset LC holds significant value as a tool. This approach could fast-track investigations into potential environmental threats in specific areas, empowering both public health officials and citizens to adopt a more holistic, region-wide strategy for safeguarding environmental health.

## Figures and Tables

**Table 1:** Number of lung cancer (LC) cases diagnosed during the period 1995–2013, the resident population during the mid-period, the difference between the observed and expected age at LC onset (residual) with the corresponding 95% Credibility Interval (CI), and the posterior probability (PP) of observing the residual, according to sex, and the selected sections of the Trieste municipality.

Selected Sections of the Municipality of Trieste	Men					Women				
	LC Incident Cases ^a^, 1995–2013	Resident Population, 30 June 2004	Residual ^b^ (Years)	(95% CI)	PP	LC Incident Cases ^a^, 1995–2013	Resident Population, 30 June 2004	Residual ^b^ (Years)	(95% CI)	PP
Coke plant										
<200 m	4	154	−3.2	(−6.7 ; 0.4)	0.003	-	166	−11.7	(−18.3 ; −5.0)	0.00001 ^c^
200–399 m	15	433	−4.5	(−8.3 ; −0.8)	0.0002 ^c^	6	480	−10.4	(−17.0 ; −3.8)	0.00001 ^c^
400–599 m	18	613	−7.7	(−11.0 ; −4.3)	0.00001 ^c^	4	717	−16.8	(−21.8 ; −11.8)	0.00001 ^c^
600–799 m										
Section A	11	303	−6.7	(−10.6 ; −2.8)	0.00001 ^c^	4	369	+0.9	(−4.9 ; 6.8)	0.70
Section B	33	1402	−5.7	(−9.0 ; −2.4)	0.00001 ^c^	12	1557	−1.8	(−7.0 ; 3.4)	0.14
Section C	-	-	-	-	-	-	-	-	-	-
800–1199 m										
Section D	36	1214	−2.7	(−7.0 ; −1.5)	0.02	23	1500	−16.2	(−23.5 ; −9.0)	0.00001 ^c^
Section E	60	2765	−0.7	(−4.1 ; 2.7)	0.25	30	3054	−5.6	(−10.9 ; −0.3)	0.0008 ^c^
Section F	5	571	−4.0	(−7.2 ; −0.8)	0.00001 ^c^	-	505	−8.4	(−13.4 ; −3.4)	0.000001 ^c^
1200–1599 m										
Section I	14	729	+1.4	(−1.8 ; 4.7)	0.92	8	842	−3.9	(−9.2 ; 1.3)	0.009
Section J	48	1474	−1.7	(−5.2 ; 1.8)	0.07	24	1726	−4.9	(−10.9 ; 1.2)	0.006
Section H	-	272	−3.9	(−7.7 ; −0.1)	0.001	-	-	-	-	-
Section G	89	2300	−4.1	(−7.7 ; −0.6)	0.0002 ^c^	35	2749	−6.7	(−13.1 ; −0.3)	0.0008 ^c^
Shipyard	-	73	−4.4	(−9.1 ; 0.2)	0.0017	-	-	-	-	-
Industrial area	287	10929	−3.0	(−6.4 ; 0.4)	0.003	108	11940	−5.3	(−10.7 ; 0.0)	0.0013
Section 3B	8	2741	−7.6	(−10.8 ; −4.3)	0.00001 ^c^	-	319	−11.6	(−16.4 ; −6.7)	0.00001 ^c^
Section 3B.1	49	1842	+1.0	(−2.3 ; 4.3)	0.83	20	2031	−8.6	(−14.1 ; −3.1)	0.00001 ^c^
Section 3B.2	46	2065	−3.4	(−6.7 ; 0.0)	0.001	13	2347	−6.3	(−11.8 ; −0.9)	0.0002 ^c^

^a^ LC Cases ≤ 3 not reported; ^b^ Residual in years: Difference between the observed and the expected median age at LC onset (negative value: early onset, positive value: late onset). ^c^ PP that were considered statistically significant after the Bonferroni correction (probability threshold = 0.00125 in men and 0.00143 in women).

## Data Availability

The study data are available under restricted access due to patient confidentiality and privacy concerns. Access can be obtained upon request to the Director of the Friuli Venezia Giulia Cancer Registry and to the Director of SC Pianificazione, Programmazione e Controllo Direzionale, Azienda Regionale di Coordinamento per la Salute, Udine, Italy. The code was based on a simple linear regression in Winbugs. No custom code or mathematical algorithm that was central to the conclusions was used in this study. The priors used were: alpha~dnorm (0.0, 1.0 × 10^−6^); beta~dnorm (0.0, 1.0 × 10^−6^); tau~dgamma (0.5, 0.01).

## References

[1] IARC Working Group on the Evaluation of Carcinogenic Risks to Humans (2016). Outdoor Air Pollution.

[2] Loomis D., Grosse Y., Lauby-Secretan B., El Ghissassi F., Bouvard V., Benbrahim-Tallaa L., Guha N., Baan R., Mattock H., Straif K. (2013). The carcinogenicity of outdoor air pollution. Lancet Oncol..

[3] Neutra R.R. (1990). Counterpoint from a cluster buster. Am. J. Epidemiol..

[4] Rothman K.J. (1990). A sobering start for the cluster busters’ conference. Am. J. Epidemiol..

[5] Pope C.A. (2000). Epidemiology of fine particulate air pollution and human health: Biologic mechanisms and who’s at risk?. Environ. Health Perspect..

[6] Samet J.M. (2011). The Clean Air Act and health—A clearer view from 2011. N. Engl. J. Med..

[7] Greenland S. (2012). Underestimating effects: Why causation probabilities need to be replaced in regulation, policy, and the law. Bull. At. Sci..

[8] Yatkin S., Gerboles M., Belis C.A., Karagulian F., Lagler F., Barbiere M., Borowiak A. (2020). Representativeness of an air quality monitoring station for PM_2.5_ and source apportionment over a small urban domain. Atmos. Pollut. Res..

[9] Krammer P., Kvassay M., Mojžiš J., Kenyeres M., Očkay M., Hluchý L., Pavlov Ľ., Skurčák Ľ. (2022). Using Satellite Imagery to Improve Local Pollution Models for High-Voltage Transmission Lines and Insulators. Future Internet.

[10] Morelli X., Rieux C., Cyrys J., Forsberg B., Slama R. (2016). Air pollution, health and social deprivation: A fine-scale risk assessment. Environ. Res..

[11] Nagl C., Spangl W., Buxbaum I. (2019). Sampling points for air quality. Scientific and Quality of Life Policies Directorate-General for Internal Policies.

[12] Pike M.C., Doll R. (1965). Age at onset of lung cancer: Significance in relation to effect of smoking. Lancet.

[13] Robins J.M., Greenland S. (1989). Estimability and estimation of excess and etiologic fractions. Stat. Med..

[14] Esteve J., Benhamou E., Raymond L. (1994). Statistical methods in cancer research. Volume IV. Descriptive epidemiology. IARC Sci. Publ..

[15] Brenner H., Gefeller O., Greenland S. (1993). Risk and rate advancement periods as measures of exposure impact on the occurrence of chronic diseases. Epidemiology.

[16] Burnet N.G., Jefferies S.J., Benson R.J., Hunt D.P., Treasure F.P. (2005). Years of life lost (YLL) from cancer is an important measure of population burden—And should be considered when allocating research funds. Br.J. Cancer.

[17] Barbone F., Bovenzi M., Cavallieri F., Stanta G. (1995). Air pollution and lung cancer in Trieste, Italy. Am. J. Epidemiol..

[18] Barbone F., Bovenzi M., Cavallieri F., Stanta G. (1997). Cigarette smoking and histologic type of lung cancer in men. Chest.

[19] Biggeri A., Barbone F., Lagazio C., Bovenzi M., Stanta G. (1996). Air pollution and lung cancer in Trieste, Italy: Spatial analysis of risk as a function of distance from sources. Environ. Health Perspect..

[20] Osservatorio Ambiente e Salute, Friuli Venezia Giulia (2014). Stato di Salute della Popolazione Residente nei Pressi del sito di Interesse Nazionale “Ferriera di Servola”: Valutazione della Mortalità e della Frequenza dei Tumori nei Comuni di Trieste e Muggia. https://www.isprambiente.gov.it/files/temi/ambiente-salute-e-societa/ValutazionemortalitcomuniTriesteMuggia.pdf.

[21] Bidoli E., Barbone F., Collarile P., Valent F., Zanier L., Daris F., Gini A., Birri S., Serraino D. (2015). Residence in Proximity of an Iron Foundry and Risk of Lung Cancer in the Municipality of Trieste, Italy, 1995–2009. Int. J. Environ. Res. Public Health.

[22] Bray F., Colombet M., Mery L., Piñeros M., Znaor A., Zanetti R., Ferlay J., Lyon F.R. (2017). Cancer Incidence in Five Continents.

[23] Bidoli E., Lamaj E., Angelin T., Forgiarini O., De Santis E., Serraino D. (2021). Linearity of Age at Cancer Onset Worldwide: 25-Year Population-Based Cancer Registry Study. Cancers.

[24] Bidoli E., Virdone S., Hamdi-Cherif M., Toffolutti F., Taborelli M., Panato C., Serraino D. (2019). Worldwide Age at Onset of Female Breast Cancer: A 25-Year Population-Based Cancer Registry Study. Sci. Rep..

[25] Newbold P., Carlson W., Torne B. (2010). Statistics for Business and Economics.

[26] Hill W., Lim E.L., Weeden C.E., Lee C., Augustine M., Chen K., Kuan F.-C., Marongiu F., Evans E.J., Moore D.A. (2023). Lung adenocarcinoma promotion by air pollutants. Nature.

[27] Malhotra J., Malvezzi M., Negri E., La Vecchia C., Boffetta P. (2016). Risk factors for lung cancer worldwide. Eur. Respir. J..

[28] Risk Factors Collaborators G.B.D. (2020). Global burden of 87 risk factors in 204 countries and territories, 1990–2019: A systematic analysis for the Global Burden of Disease Study 2019. Lancet.

[29] European Environment Agency (2020). Air Quality in Europe—2020 Report.

[30] Saltelli A., Bammer G., Bruno I., Charters E., Di Fiore M., Didier E., Nelson Espeland W., Kay J., Lo Piano S., Mayo D. (2020). Five ways to ensure that models serve society: A manifesto. Nature.

[31] Lelieveld J., Pozzer A., Poschl U., Fnais M., Haines A., Munzel T. (2020). Loss of life expectancy from air pollution compared to other risk factors: A worldwide perspective. Cardiovasc. Res..

[32] Berry G. (2007). Relative risk and acceleration in lung cancer. Stat. Med..

